# Reduced splenic uptake on ^68^Ga-Pentixafor-PET/CT imaging in multiple myeloma - a potential imaging biomarker for disease prognosis

**DOI:** 10.7150/thno.75847

**Published:** 2022-08-08

**Authors:** Sabrina Kraus, Philipp Klassen, Malte Kircher, Alexander Dierks, Stefan Habringer, Alexander Gäble, Klaus Martin Kortüm, Niels Weinhold, Valëza Ademaj-Kospiri, Rudolf A Werner, Andreas Schirbel, Andreas K Buck, Peter Herhaus, Hans-Jürgen Wester, Andreas Rosenwald, Wolfgang A Weber, Hermann Einsele, Ulrich Keller, Leo Rasche, Constantin Lapa

**Affiliations:** 1 Department of Internal Medicine II, University Hospital of Würzburg, Würzburg, Germany.; 2 Department of Nuclear Medicine, University Hospital of Würzburg, Würzburg, Germany.; 3 Nuclear Medicine, Faculty of Medicine, University of Augsburg, Augsburg, Germany.; 4 Department of Hematology, Oncology and Cancer Immunology, Campus Benjamin Franklin, Charité - Universitätsmedizin Berlin, Corporate Member of Freie Universität Berlin and Humboldt-Universität zu Berlin, Berlin, Germany.; 5 Department of Internal Medicine V, Heidelberg University Hospital, Heidelberg, Germany.; 6 Department of Nuclear Medicine, Klinikum rechts der Isar, Technische Universität München, Munich, Germany.; 7 The Russell H. Morgan Department of Radiology and Radiological Science, Johns Hopkins University School of Medicine, Baltimore, MD, USA.; 8 Technical University Munich, School of Medicine, Klinikum rechts der Isar, Clinic and Policlinic for Internal Medicine III, Munich, Germany.; 9 Pharmaceutical Radiochemistry, Technical University of Munich, Munich, Germany.; 10 Institute of Pathology, University of Würzburg, Würzburg, Germany.

**Keywords:** multiple myeloma, ^68^Ga-Pentixafor-PET/CT, CXCR4, molecular imaging, spleen

## Abstract

Beyond being a key factor for tumor growth and metastasis in human cancer, C-X-C motif chemokine receptor 4 (CXCR4) is also highly expressed by a number of immune cells, allowing for non-invasive read-out of inflammatory activity. With two recent studies reporting on prognostic implications of the spleen signal in diffusion-weighted magnetic resonance imaging in patients with plasma cell dyscrasias, the aim of this study was to correlate splenic ^68^Ga-Pentixafor uptake in multiple myeloma (MM) with clinical parameters and to evaluate its prognostic impact.

**Methods:** Eighty-seven MM patients underwent molecular imaging with ^68^Ga-Pentixafor-PET/CT. Splenic CXCR4 expression was semi-quantitatively assessed by peak standardized uptake values (SUV_peak_) and corresponding spleen-to-bloodpool ratios (TBR) and correlated with clinical and prognostic features as well as survival parameters.

**Results:** ^68^Ga-Pentixafor-PET/CT was visually positive in all MM patients with markedly heterogeneous tracer uptake in the spleen. CXCR4 expression determined by ^68^Ga-Pentixafor-PET/CT corresponded with advanced disease and was inversely associated with the number of previous treatment lines as compared to controls or untreated smouldering multiple myeloma patients (SUV_peak_Spleen 4.06 ± 1.43 vs. 6.02 ± 1.16 vs. 7.33 ± 1.40; *P <* 0.001). Moreover, reduced splenic ^68^Ga-Pentixafor uptake was linked to unfavorable clinical outcome. Patients with a low SUV_peak_Spleen (<3.35) experienced a significantly shorter overall survival of 5 months as compared to 62 months in patients with a high SUV_peak_Spleen >5.79 (*P <* 0.001). Multivariate Cox analysis confirmed SUV_peak_Spleen as an independent predictor of survival (HR 0.75; *P =* 0.009).

**Conclusion:** These data suggest that splenic ^68^Ga-Pentixafor uptake might provide prognostic information in pre-treated MM patients similar to what was reported for diffusion-weighted magnetic resonance imaging. Further research to elucidate the underlying biologic implications is warranted.

## Introduction

Multiple myeloma (MM) is a biologically heterogeneous disease characterized by the uncontrolled clonal proliferation of plasma cells predominantly within the bone marrow (BM) 1. Both spatial and temporal heterogeneity and extramedullary involvement are recognized as additional factors to the complexity of this disease 2-4. Recent advances in the diagnosis and treatment of MM have highlighted the importance of imaging methods not only in disease localization and staging, but also in prognostic stratification and response assessment 5. The value of positron emission tomography/computed tomography (PET/CT) with ^18^F-labelled fluorodeoxyglucose (^18^F-FDG) has been widely investigated in varying clinical conditions and is recognized as a useful tool in the management of patients with MM 6-11. However, ^18^F-FDG PET/CT also has some limitations, such as lower sensitivity in detecting bone marrow involvement in MM patients, so alternative tracers have been investigated 12-17.

C-X-C motif chemokine receptor 4 (CXCR4) is a rising ligand in molecular imaging. Beyond its physiologic role in homing of hematopoietic stem (HSCs) and immune cells 18, 19, receptor overexpression could be demonstrated in more than 30 different types of cancer including multiple myeloma, diffuse large B-cell lymphoma, breast cancer and small cell lung cancer 20-26 and could be identified as an adverse prognostic factor 20. In MM, the interplay between receptor and its ligand stromal derived factor 1 (SDF-1α/CXCL12) serves as a stimulus of plasma cell proliferation 27.

^68^Ga-Pentixafor, a synthetic ligand of CXCR4, is a promising tool for *in vivo* positron emission tomography (PET) imaging of many solid and hematologic malignancies 25, 28-32. Noteworthy, a wide heterogeneity not only of tumor, but also spleen CXCR4 expression could be observed 31, 33.

In this context, Rasche et al. demonstrated the spleen signal in diffusion-weighted magnetic resonance imaging (DW-MRI) to be associated with tumor burden and prognosis in 295 newly diagnosed MM (NDMM) patients 34. Recently, this observation was confirmed in a Japanese study with 96 patients, reporting that loss of spleen visualization on DW-MRI imaging correlates with high tumor volume and poor prognosis 35. Interestingly, heterogeneous splenic ^68^Ga-Pentixafor uptake could also be observed in MM patients. Therefore, the aim of this study was to evaluate the prognostic impact of ^68^Ga-Pentixafor uptake in the spleen of MM patients and to correlate it with clinical data and clinical outcome.

## Materials and methods

Study approval was obtained from the local ethics committee of the University of Würzburg, Germany (213/13), and all patients signed written informed consent prior to ^68^Ga-Pentixafor-PET/CT in accordance with the Declaration of Helsinki.

### Subjects and study design

Between July 2013 and May 2018, 87 patients with pre-treated MM (median of previous treatment lines 4; range, 1-12) underwent molecular imaging with ^68^Ga-Pentixafor-PET/CT for diagnostic work-up at the University Center of Würzburg. MM was diagnosed according to the current IMWG guidelines 36. Detailed patients' characteristics are displayed in **Table 1** and **Table S2.** At the time point of ^68^Ga-Pentixafor-PET/CT scanning, patients' treatment history, M-gradient (available in n = 75/87), serum free immunoglobulin light chains (FLC; n = 75/87) and hematological parameters (all patients) were recorded. The following hematological parameters were measured (all patients): leukocyte count, lymphocyte count, neutrophil count, monocyte count, reticulocyte count, red blood cell count (RBC), platelet count (Plt), hemoglobin (Hb), and hematocrit (Hct). Serum chemistry including lactate dehydrogenase (LDH, all patients), C-reactive protein (n = 82/87), albumin (n = 85/87), creatinine (all patients) and β2-microglobulin (n = 75/87) levels were obtained. The mean time interval time between determination of laboratory values and ^68^Ga-Pentixafor-PET/CT was 7±12 days. International Staging System (ISS) stages for the day of PET imaging were calculated. Additionally, interphase molecular cytogenetics based on fluorescence *in situ* hybridization (FISH) were available in 67/87 patients. High-risk cytogenetic abnormalities were defined by the presence of del17p, t(4;14) and t(14;16). Recent bone marrow biopsy results for assessment of malignant clonal plasma cells were available in n = 28/87 subjects. The median time between bone marrow biopsy (available in 28/87 patients) to ^68^Ga-Pentixafor PET/CT scan was 11±17 days (range, 0-72 days).

Nine patients with smoldering multiple myeloma (SMM; 7 males, 2 females; mean age 64±10 years) served as treatment-naive oncologic controls. In addition, five non-oncologic patients (3 males, 2 females; mean age 59±8 years) who underwent ^68^Ga-Pentixafor-PET/CT due to suspicion of benign Conn's adenoma were included as a “healthy” comparison group. As previously described, these patients represent the most suitable control group because the exposure of healthy volunteers to unnecessary radiation cannot be justified 37.

We used the ^68^Ga-Pentixafor uptake in the spleen of all MM patients and divided the patients into 3 uptake groups (<25%, 25%-75% and >75% quantiles) to determine the threshold for increased and decreased splenic uptake, respectively. SUV_peak_Spleen of 3.35 and 5.79 were used to distinguish high (>5.79), intermediate (3.35-5.79) and low (<3.35) ^68^Ga-Pentixafor uptake in the spleen.

### Patient follow-up

In order to investigate the prognostic value of splenic ^68^Ga-Pentixafor uptake, individual patients´ outcomes were assessed in terms of overall survival (OS) using the following formula: OS = [(Date of death)-(Date of PET imaging)].

### PET/CT imaging

^68^Ga-Pentixafor was synthesized in-house using a fully GMP compliant automated synthesizer (GRP, Scintomics, Fürstenfeldbruck, Germany), as previously described 38. After injection of ^68^Ga-Pentixafor (median 127 MBq; range 43-207 MBq) all PET/CT scans were performed on a dedicated PET/CT scanner (Siemens Biograph mCT 64; Siemens Medical Solutions, Erlangen, Germany) using standard acquisition and reconstruction protocols. For anatomical correlation and attenuation correction we subsequently acquired low-dose CT scans (35mAs, 120keV, a 512 x 512 matrix, 5 mm slice thickness, increment of 30mm/s, rotation time of 0.5s, and pitch index of 0.8). PET images were reconstructed using standard parameters (HD-PET, 3 iterations, 24 subsets, Gaussian filtering: 2 mm, resolution: axial resolution: 5 mm, in-plane resolution: 4 x 4 mm²) and corrected for attenuation, dead-time, random events and scatter.

### Image analysis

PET/CT scans were visually assessed by two board-certified nuclear medicine physicians (CL and MK). First, a visual inspection of scans for elevated splenic tracer uptake was performed. For semi-quantitative analysis, standardized uptake values (SUV) for splenic uptake were determined by placing a volume of interest (VOI) with a diameter of 4 cm. The average SUV within a 10 mm circular region centered on the pixel with the highest tracer uptake was defined as SUV_peak_.

For derivation of background activity, a 15 mm ROI was placed in the center of the right atrium and mean standardized uptake values (SUV_bloodpool_) were recorded. Afterwards, a target-to-background ratio (TBR) was calculated by dividing SUV_peak_Spleen by SUV_bloodpool_. In addition, mean tracer uptake in a sphere with a diameter of 4 cm within the liver (SUV_mean_Liver) was recorded. The radiotracer concentration in the ROIs was normalized to the injected dose per kilogram of patient's body weight to derive the SUVs.

### Statistics

Statistical analyses were performed using R (version 3.6.1, R Core Team, 2019) with packages caret (version 6.0.84), rms (version 5.1-3.1), and survival (version 2.38). All results are displayed as mean ± SD or as median + range wherever appropriate. The Kaplan-Meier method was used to analyze the survival outcome of the patients. A two-tailed log-rank test was used to compare the survival outcomes between the subgroups. For bivariate correlation analyses, Spearman or Pearson correlation coefficients were calculated. A *P-*value of <0.05 was considered statistically significant.

## Results

### Splenic ^68^Ga-Pentixafor uptake levels show a great variability in MM patients

CXCR4-directed PET/CT imaging with ^68^Ga-pentixafor was visually positive in all studied MM patients (n = 87), with very heterogeneous uptake in the spleen. For assessing physiologic splenic ^68^Ga-Pentixafor uptake, five patients who underwent CXCR4-directed PET/CT due to suspicion of benign Conn's adenoma were included as a control group. MM patients with a SUV_peak_Spleen lower than 3.35 (TBR 2.11) and above 5.79 (TBR 3.51) were considered as low-expression group and high-expression group, respectively. All other subjects were classified into an intermediate group.

SUV_peak_Spleen ranged from 1.4 to 10.0 with a median of 4.5 and a median TBR of 2.97 (range, 1.3-6.2), respectively. SUV_peak_Spleen and TBRs are summarized in **Table S1**. According to the three defined uptake categories (low, intermediate, high), 22/87 subjects qualified for the low, 42/87 for the intermediate and 23/87 for the high expression group. Detailed patients' characteristics are displayed in **Table 1.**

### Reduced splenic ^68^Ga-Pentixafor uptake is associated with advanced disease stage and bone marrow insufficiency

Differences in spleen CXCR4 expression as visualized by ^68^Ga-Pentixafor-PET/CT could potentially be associated with tumor burden and hematopoietic reserve, as this was reported in previous studies investigating the spleen signal using MRI 34, 35. In detail, we investigated whether splenic uptake was associated with ISS, presence of extramedullary disease (EMD), or the number of focal lesions. We found ^68^Ga-Pentixafor uptake of the spleen to be negatively associated with ISS stage at the time point of PET imaging (r = -0.409, *P <* 0.001), and percentage of malignant plasma cells in the bone marrow (r = -0.534, *P =* 0.003). Furthermore, presence of EMD was negatively correlated with CXCR4 expression (r = -0.352, *P =* 0.001). Regarding markers of acute inflammation, both serum ferritin (r = -0.462, *P =* 0.001) as well as C-reactive protein levels (r = -0.287, *P =* 0.009) were inversely correlated with the splenic PET signal (**Table 2**).

In contrast, splenic ^68^Ga-pentixafor uptake was not correlated with myeloma CXCR4 positivity of the scan (r = -0.154, *P =* 0.154), high-risk cytogenetics (r = -0.127, *P =* 0.306), the serum level of the involved free light chain (r = -0.115, *P =* 0.325), the pattern of myeloma bone marrow burden (focal vs. diffuse vs. focal-on-diffuse; r = -0.017, *P =* 0.873) or the number of PET-positive focal lesions (r = -0.153, *P =* 0.156). In addition, we correlated ^68^Ga-pentixafor uptake in MM lesions and splenic ^68^Ga-pentixafor uptake and could not find a significant association (r_s_=-0.15; *P =* 0.161).

We hypothesized that differences in spleen CXCR4 expression as measured by ^68^Ga-Pentixafor-PET/CT could also be associated with hematopoietic insufficiency. Hence, we evaluated hematological parameters of all patients on the day of imaging and correlated SUV_peak_Spleen with peripheral leukocyte count (/nl), hemoglobin levels (g/dl) and peripheral platelet count (/µl). Serum chemistry including lactate dehydrogenase (LDH), albumin, creatinine, and β2-microglobulin was also assessed. When investigating hematological parameters, we could observe a significant positive correlation of spleen ^68^Ga-Pentixafor uptake with higher counts for hemoglobin (r = 0.388, *P <* 0.001), peripheral thrombocytes (r = 0.524, *P <* 0.001), and peripheral leukocytes (r = 0.669, *P <* 0.001). Together, our observations suggest that a reduced splenic signal is mainly associated with parameters reflecting tumor burden or hematopoietic insufficiency rather than with specific tumor features. Individual values for the various laboratory parameters are displayed in **Table 2**.

### Dynamics of splenic ^68^Ga-Pentixafor uptake levels during the course of MM disease

**Figure 1:**
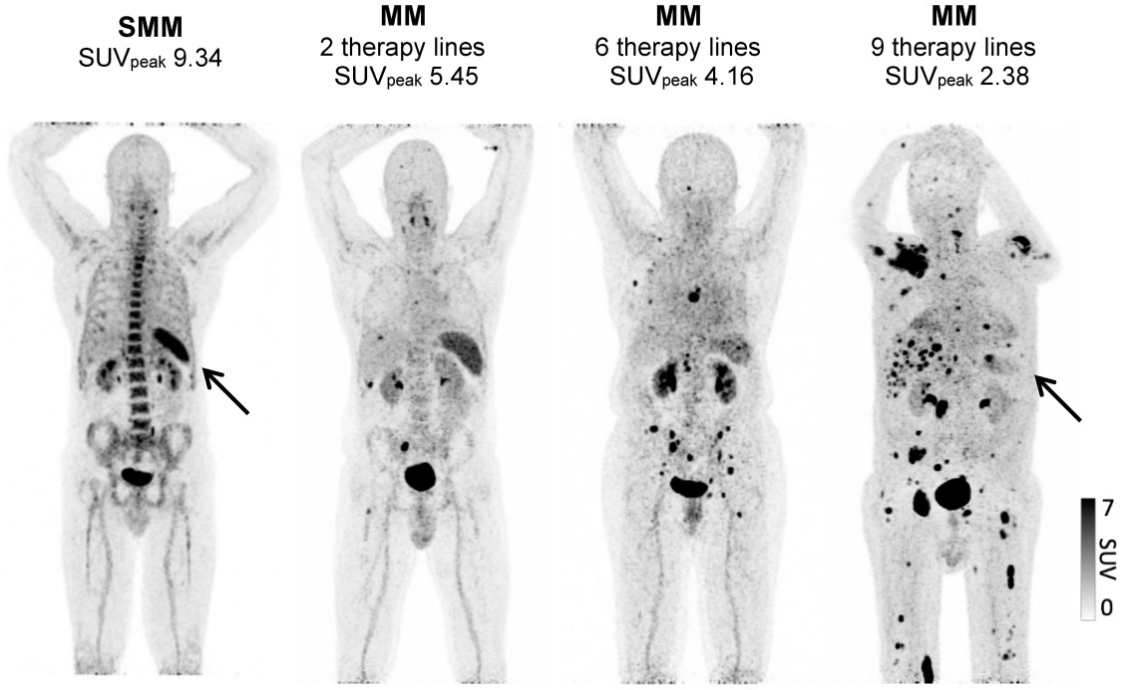
** Spleen ^68^Ga-Pentixafor uptake during MM therapy.** Association between splenic ^68^Ga-Pentixafor uptake at baseline and during disease progression with increasing lines of therapy.** A:** Representative ^68^Ga-Pentixafor-PET/CT images for patients with plasma cell disorders with increasing lines of therapies are shown. While the patient with smoldering myeloma (SMM) presents with intense tracer uptake, the splenic signal gradually continues to decrease with increasing lines of therapy.

**Figure 2:**
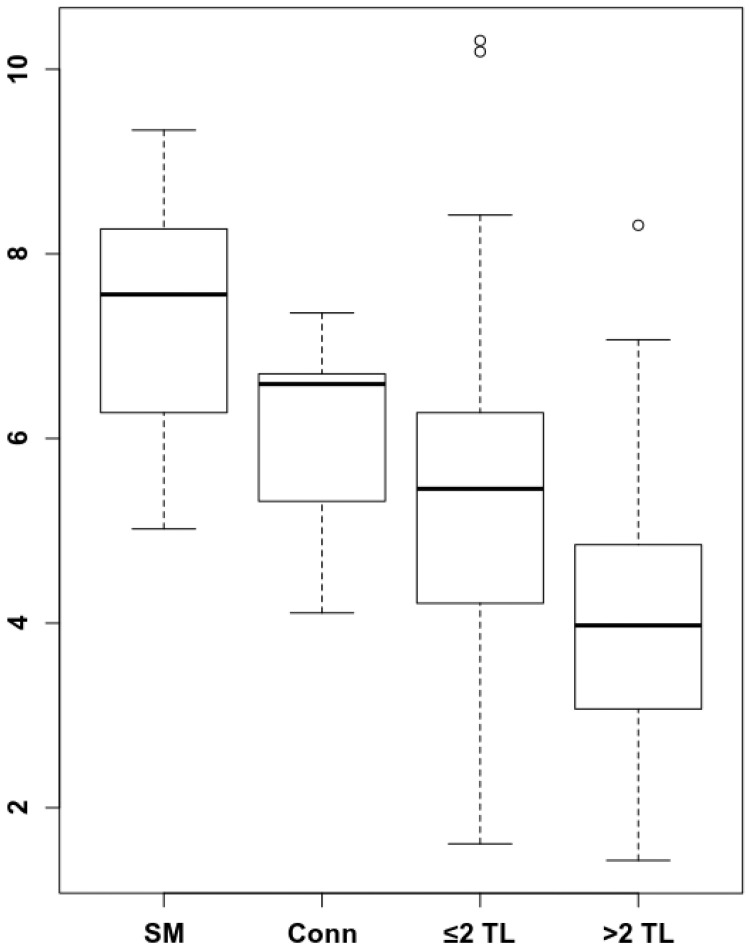
** Spleen ^68^Ga-Pentixafor uptake during MM therapy.** The association between spleen ^68^Ga-Pentixafor uptake and therapy lines is depicted (SMM 7.33 ± 1.40; Conn 6.02 ± 1.16; ≤2TL 5.48 ± 1.93; >2TL 4.06 ± 1.43). *P*-value °<0.05, °°<0.01; °°°<0.001.

To understand by which parameters the spleen signal is altered and how this correlates with remaining survival time, we analyzed whether there are longitudinal dynamics in splenic ^68^Ga-Pentixafor uptake and whether there is a correlation with extramedullary hematopoiesis or tumor burden. The intensity of the splenic ^68^Ga-Pentixafor uptake shows dynamic changes during the course of MM disease. Patients with SMM show higher ^68^Ga-Pentixafor uptake in the spleen with an SUV_peak_Spleen of 7.33 ± 1.40 compared to controls with an SUV_peak_Spleen of 6.02 ± 1.16. Further, we hypothesized that systemic treatment could potentially influence CXCR4 receptor expression. Hence, we compared SMM patients who had not undergone treatment before imaging to prior systemically treated MM patients. We detected that spleen ^68^Ga-Pentixafor uptake decreases with treatment duration and an increasing number of treatment lines **(Figure 1)**. In patients receiving one to two lines of systemic treatment, SUV_peak_Spleen was 5.48 ± 1.93, and in patients receiving more than two lines of treatment, this value decreased to 4.06 ± 1.43 (**Figure 2**, *P <* 0.001).

### Spleen ^68^Ga-Pentixafor uptake is associated with clinical outcome in MM patients

**Figure 3:**
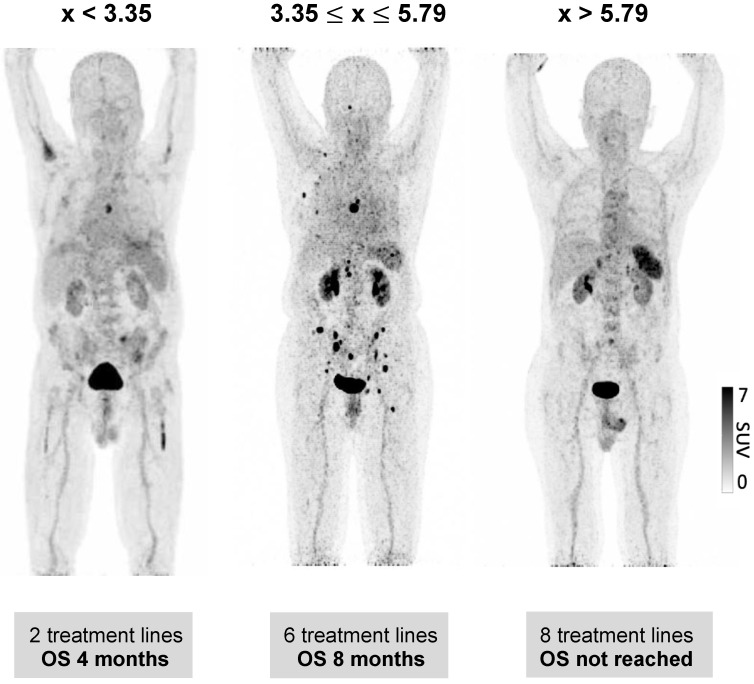
** Prognostic value of splenic ^68^Ga-Pentixafor uptake.** Stratification of MM patients in three prognostic groups using SUV_peak_Spleen: I. <3.35; II. 3.35-5.79; III. >5.79.

**Figure 4:**
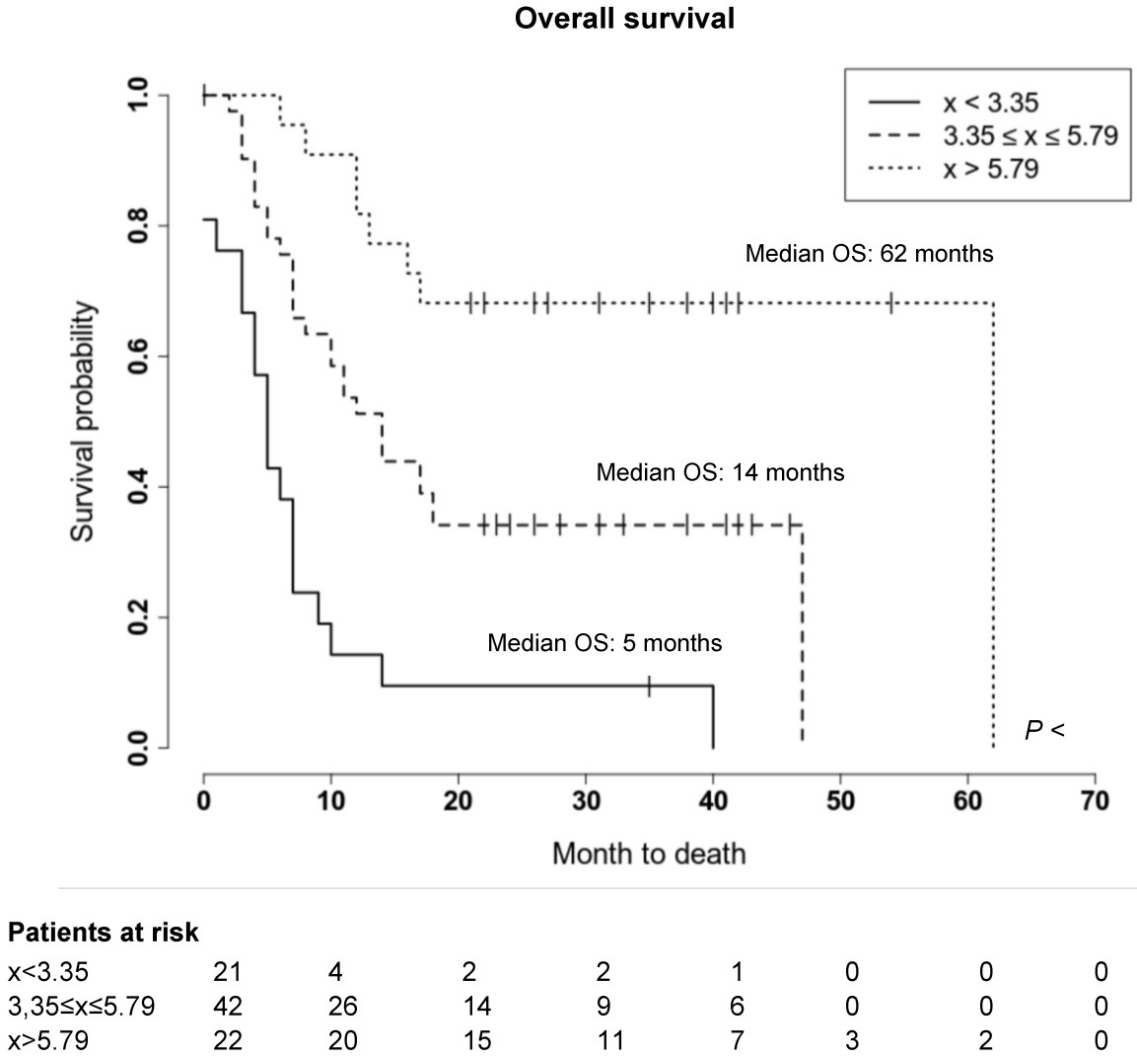
** Prognostic value of splenic ^68^Ga-Pentixafor uptake.** Overall survival of MM patients stratified by SUV_peak_Spleen in three prognostic subgroups. Patients with a SUV_peak_Spleen >5.79 showed a significantly longer overall survival (*P <* 0.001). Given are the cumulated survival (y-axis) and the overall survival (in months; x-axis).

We further evaluated whether the observed wide variability of spleen CXCR4 expression is also associated with clinical outcome of MM patients. Indeed, a significant correlation between splenic uptake and overall survival (OS) could be observed. Thus, we compared OS by stratifying all patients according to the median SUV_peak_Spleen into three groups of low, intermediate and high spleen CXCR4 expression **(Figure 3)**. While in the group with a high SUV_peak_Spleen >5.79 (TBR >3.51), the OS of MM patients was 62 months, patients in the group with an intermediate SUV_peak_Spleen of 3.35 to 5.79 (TBR 2.11-3.50) and in the group with a low SUV_peak_Spleen <3.35 (TBR <2.11) showed a significantly lower median OS of 14 and 5 months, respectively (**Figure 4**, *P <* 0.001). The association remained significant in multivariate Cox regression analysis (including age, EMD, number of PET-positive focal lesions, anemia, ISS stage, elevated CRP level and number of prior treatment lines), confirming that the splenic ^68^Ga-Pentixafor signal can provide independent prognostic information in MM (HR 0.75, P = 0.009; **Table 3**).

In contrast to splenic ^68^Ga-Pentixafor uptake, mean SUV of the liver was not associated with clinical outcome (**Table S1**; **Figure S1**).

## Discussion

Here, we present the first study investigating splenic uptake of ^68^Ga-Pentixafor in MM patients. Previously, we and others have already demonstrated the feasibility of CXCR4-directed PET imaging as a suitable tool for both non-invasive detection of MM lesions as well as patient identification for CXCR4-directed therapy with pilot studies suggesting the feasibility of CXCR4-directed radioligand therapy as a novel treatment approach for MM 29. In this study, we show for the first time that the intensity of ^68^Ga-Pentixafor uptake in the spleen, an organ which is frequently overlooked during myeloma examinations, is strongly associated with OS and with parameters that indicate extramedullary hematopoiesis (EMH).

Noteworthy, our results are in line with a recent report by the Little Rock group who reported on prognostic implications of the diffusion-weighted magnetic resonance imaging-derived restriction level of the spleen in 295 newly diagnosed MM patients 34. The authors hypothesized that the spleen signal might prove a promising proxy for tumor load in MM and also be associated with response and prognosis. Since absence of spleen signal was highly correlated with higher myeloma burden in terms of malignant bone marrow plasma cells, they postulated that EMH might be the underlying biologic mechanism for this phenomenon, assuming that MM cells crowd out other hematopoietic cells in the BM. This was questioned by a recent Japanese study of 96 NDMM patients investigating the clinical significance of loss of spleen visualization on DW-MRI. They could also show that loss of spleen visualization is associated with high tumor burden and poor prognosis 35.

In our cohort of pre-treated MM patients, we were able to corroborate these MRI findings with ^68^Ga-Pentixafor uptake of the spleen being negatively associated with ISS stage at the time point of PET imaging, percentage of malignant plasma cells in the bone marrow as well as presence of extramedullary disease at the time point of PET imaging. Of note, also disease duration and intensity of previous treatment could be correlated with the splenic PET signal: Whereas untreated patients with SMM demonstrated the highest ^68^Ga-Pentixafor uptake in the spleen with an SUV_peak_ of 7.33 ± 1.40 (as compared to controls with an SUV_peak_Spleen of 6.02 ± 1.16), tracer accumulation diminished with an increasing number of treatment lines. Since higher blood counts were associated with higher SUV_peak_Spleen, one might hypothesize that the spleen might also serve as a biomarker of disease stage. Having stratified patients into a high, intermediate and low CXCR4 expression group, respectively, reduced splenic ^68^Ga-Pentixafor uptake was significantly associated with shorter OS, independent of the number of prior treatment lines.

The underlying biology of the observed phenomenon is still not elucidated yet as spleen biopsies could not be obtained in the recent study and analysis using autopsy specimens is of limited value given the high autolytic activity within the spleen. One potential explanation is that reduced splenic CXCR4 expression is a marker of reduced EMH. For example, Cao and colleagues observed in an experiment with luciferase-labeled HSCs in mice that the most frequent site of initial engraftment was the spleen and that signal intensity steadily increased over the first 6 weeks after transplant. They proposed that - after myeloablation - the spleen is able to convert from a predominantly lymphoid into a hematopoietic tissue 39, 40. Additionally, Miwa et al. were able to show that in EMH conditions CXCL12 expression in human spleens is higher than in an environment without EMH 41, supporting our thesis of monitoring hematopoietic constitution via CXCR4-directed imaging.

Another possibility derived from magnetic resonance imaging is that the reduced signal originates from iron overload in the spleen due to red blood cell transfusions. Of note, we were able to record a significant inverse correlation between serum ferritin levels and the splenic ^68^Ga-Pentixafor uptake, it is thus conceivable that the reduction of the measurable signal might be due to the displacement of CXCR4 positive cells. In another sense, ferritin also acts as an acute-phase protein, thus elevated serum levels might indicate inflammatory activity. Consistent with this, C-reactive protein - another proven adverse prognostic factor in MM 25, 26 - was also found to have a significant inverse correlation with SUV_peak_ of the spleen in our cohort.

In contrast, the theory that myeloma plasma cells might directly modify the spleen signal by infiltrating this organ is rather unlikely since we did not observe any correlation between myeloma CXCR4 positivity and SUV_peak_ of the spleen. Since no relevant correlation between overall survival and radiotracer accumulation in the liver could be observed in our cohort, a tumor sink effect as a major contributor to the observed phenomenon seems unlikely. Along these lines, a recent publication in 90 patients with histologically proven solid cancers undergoing CXCR4-targeted PET/CT 42 also reported no relevant tumor sink effect. Further research to gain a better understanding of the biologic processes and cell types responsible for the present observation is highly needed.

Beyond hematologic malignancies, Lewis at al. recently analyzed splenic ^68^Ga-Pentixafor uptake in 145 patients with various solid cancers 33. Although platelet counts and/or leukocyte counts correlated positively with spleen ^68^Ga-Pentixafor uptake in non-small cell lung cancer, small cell lung cancer and neuroendocrine tumors, no association between (increased or decreased) spleen ^68^Ga-Pentixafor uptake and survival or prior systemic treatment could be observed. Whereas CXCR4 signaling and consequently differences in spleen ^68^Ga-Pentixafor uptake might be more relevant in MM patients than in solid cancers, no firm conclusions can be drawn yet. Further studies need to clarify the mechanism underlying the highly variable spleen ^68^Ga-Pentixafor uptake of MM patients to better assess both clinical and therapeutic consequences.

This retrospective study suffers from several limitations. First, we included a rather heterogeneous population of pre-treated MM patients. 84/87 patients received at least one stem cell transplantation, but the number of previous therapy varied to up to 12 different lines of treatment. Secondly, lacking physiologic uptake values of ^68^Ga-Pentixafor for the spleen in a healthy population, we used the data from a small collective of patients suffering from Conn´s disease to determine decreased and increased uptake. Nonetheless, these values performed well in distinguishing three groups with highly significant different outcomes.

## Conclusions

Our data suggest that CXCR4 expression in the spleen might provide prognostic information in pre-treated MM patients. Further research to elucidate the underlying biologic implications is warranted.

## Supplementary Material

Supplementary figure and tables.

## Figures and Tables

**Table 1:** Patients' characteristics

Clinical characteristics	No. of Patients (%), Total (n = 87)
**Age**	
> 65 years	13 (14.9%)
≤ 65 years	74 (85.1%)
**Sex**	
Male	60 (69.0%)
Female	27 (31.0%)
**Histologic subtype**	
IgG	41 (47.1%)
IgA	20 (23.0%)
IgD	0 (0%)
***Light chain only***	
Lamda	13 (14.9%)
Kappa	12 (13.8%)
Non-secretory	1 (1.1%)
**Cytogenetic high risk abnormalities on FISH**	
del(17p)	12 (13.8%)
t(4;14)	10 (11.5%)
t(14;16)	1 (1.1%)
Cytogenetics not available	20 (23.0%)
**ISS (at PET/CT imaging)**	
I	26 (30.0%)
II	39 (44.8%)
III	10 (11.5%)
ISS not available	12 (13.8%)
**R-ISS (ISS (at PET/CT imaging)**	
I	10 (11.5%)
II	33 (37.9%)
III	24 (27.6%)
R-ISS not available	20 (23.0%)
Extramedullary disease	23 (26.4%)
Prior high-dose therapy with autologous SCT	84 (97.0%)
**Bone marrow plasma cell percentage**	38.0 (1-90%)
Median (range), %	Histology available in 28/97 patients
**Laboratory finding**	
Anemia (<13.5g/dl)	74 (85.0%); median 11.1 (range 7.0-15.2)
Elevated LDH (>250 U/I)	34 (39.1%); median 281 (range 113-1189)
Hypercalcemia (>2.6 mmol/l)	2 (2.3%); median 2.3 (range 1.5-3.2)
Elevated β2-microglobulin (>2.4 mg(L)	40 (46.0%); median 4.5 (range 1.1-69)
***β2-microglobulin not available n = 12/87***	
Elevated CRP (>1 mg/dl)	26 (30.0%); median 1.4 (range 0.0-22.0)
Elevated creatinine (>1.09 mg/dl)	37 (43.0%); median 1.2 (range 0.6-8.7)

SCT: stem cell transplantation; ISS: international staging system; FISH: fluorescence staging system; CRP: C-reactive protein; LDH: lactate dehydrogenase.

**Table 2:** Correlation with SUV_peak_Spleen

Variable	n	Pearson			Spearman	
		r	*P*	CI	r	*P*
Age	87	- 0.120	0.270	-0,322, 0.093	-0.101	0.352
Number of lesions	87	-0.153	0.156	-0.353, 0.059	-0.152	0.161
Extramedullary disease	87	-0,337	0.001	-0.511, -0.136	-0.352	0.001
Number of therapy lines before PET/CT	86	-0.388	<0.001	-0.555, -0.192	-0.437	<0.001
Histology (% plasmocytes in bone marrow)	28	-0.534	0.003	-0.757, -0.202	-0.431	0.022
ISS at point of PET/CT	75	-0.409	<0.001	-0.576, -0.209	-0.439	<0.001
Leucocytes	87	0.315	0.003	0.112, 0.493	0.388	<0.001
Hemoglobin	87	0.522	<0.001	0.350, 0.660	0.524	<0.001
Thrombocytes	87	0.547	<0.001	0.380, 0.679	0.669	<0.001
Calcium	85	0.131	0.234	-0.085, 0.334	0.185	0.090
Creatinine	87	-0.020	0.857	-0.229, 0.192	0.039	0.717
LDH	87	-0.199	0.065	-0.393, 0.012	-0.143	0.185
Albumin	85	0.450	<0.001	0.262, 0.605	0.449	<0.001
Ferritin	50	-0.462	0.001	-0.656, -0.210	-0.623	<0.001
CRP	82	-0.287	0.009	-0.475, -0.075	-0.369	0.001
ß2-microglobulin	75	-0.197	0.091	-0.405, 0.032	-0.407	<0.001
High risk cytogenetics	67	-0.127	0.306	-0.356, 0.117	-0,128	0.303

ISS international staging system; CRP C-reactive protein; LDH lactate dehydrogenase.

**Table 3:** Results of multivariate cox regression analysis assessing relationship between SUV_peak_Spleen, clinical parameters and overall survival

Variable	Hazard Ratio	95% Confidence interval	*P*-value
SUV_peak_Spleen	0.75	0.68-0.84	0.009*
Age	1.00	0.98-1.02	0.907
Number of PET-positive lesions	1.16	1.02-1.31	0.249
Extramedullary disease	1.00	0.67-1.48	0.995
Number of prior treatment lines	1.09	1.03-1.16	0.123
Anemia	1.77	1.06-2.98	0.269
ISS stage	1.42	1.07-1.89	0.215
Elevated CRP level	0.79	0.40-1.56	0.730

*A *P*-value <0.05 was considered significant.

## Abbreviations

CXCR4: C-X-C motif chemokine receptor 4
MM: multiple myeloma
SUV_peak_: standardized uptake values
BM: bone marrow
PET/CT: positron emission tomography/computed tomography
^18^F-FDG: ^18^F-labelled fluorodeoxyglucose
HSCs: hematopoietic stem cells
SDF-1α: stromal derived factor 1
CXCL12: C-X-C motif chemokine ligand 12
DW-MRI: diffusion-weighted magnetic resonance imaging
NDMM: newly diagnosed MM
RBC: red blood cell count
Plt: platelet count
Hb: hemoglobin
Hct: hematocrit
LDH: lactate dehydrogenase
FISH: fluorescence *in situ* hybridization
SMM: smoldering multiple myeloma
OS: overall survival
EMD: extramedullary disease
VOI: volume of interest
TBR: target-to-background ratio
EMH: extramedullary hematopoiesis
