# Supplementary material for: Reduced splenic uptake on 68Ga-Pentixafor-PET/CT imaging in multiple myeloma - a potential imaging biomarker for disease prognosis
Source: Theranostics. 2022 Aug 8;12(13):5986–94. doi: 10.7150/thno.75847 (PMC9373803; doi:10.7150/thno.75847)
Supplement: Supplementary file 1 — Supplementary figure and tables. [file thnov12p5986s1.pdf]

SUPPLEMENTARY TABLE S1: Patients` characteristics and imaging results

Patient No. #	MM Type	Prior therapy lines	CXCR4 SUV_{peak}Spleen	CXCR4 SUV_{mean}bloodpool	TBR	CXCR4 SUV_{mean}Liver
#1	light chain	2	10.31	1.66	6.21	1.41
#2	light chain	2	10.19	1.95	5.23	1.76
#3	IgG	1	8.42	1.55	5.43	0.94
#4	IgG	4	8.31	1.82	4.57	1.03
#5	light chain	1	8.14	1.87	4.35	1.22
#6	light chain	1	8	3.11	2.57	2.4
#7	IgA	2	7.23	1.77	4.08	1
#8	light chain	2	5.8	1.31	4.43	1.44
#9	light chain	3	7.07	2.28	3.10	1.41
#10	IgG	6	6.83	2.24	3.05	1.3
#11	IgG	4	6.57	1.61	4.08	1.37
#12	light chain	1	6.55	2.36	2.78	1.7
#13	IgG	2	6.34	2.04	3.11	1.72
#14	IgG	1	6.31	1.63	3.87	1.11
#15	IgG	2	6.25	2.7	2.31	1.76
#16	light chain	4	6.24	1.77	3.53	1.54
#17	IgG	1	6.23	2.02	3.08	1.22
#18	IgG	2	6.22	1.44	4.32	1.02
#19	IgG	1	6.15	1.94	3.17	1.1
#20	IgG	2	6.1	1.69	3.61	1.29
#21	light chain	2	5.92	2.45	2.42	1.33
#22	IgG	1	5.83	1.28	4.55	1.14
#23	IgA	4	5.77	1.84	3.14	0.76
#24	IgA	3	5.73	1.54	3.72	1.74
#25	IgG	3	5.65	1.32	4.28	1.63
#26	IgG	4	5.6	1.76	3.18	1.48
#27	light chain	1	5.46	1.27	4.30	0.58
#28	IgG	3	5.45	1.37	3.98	1.26
#29	light chain	5	5.11	1.08	4.73	0.72
#30	igG	4	5.11	1.6	3.19	0.73
#31	IgA	2	4.99	2.2	2.27	1.09
#32	IgG	2	4.91	1.05	4.68	0.92
#33	IgG	1	4.89	1.57	3.11	1
#34	igG	3	4.87	1.41	3.45	1.22
#35	IgG	4	4.85	1.96	2.47	1.09
#36	IgA	2	4.76	1.66	2.87	0.74
#37	light chain	2	4.74	2.14	2.21	1.22
#38	light chain	5	4.64	1.84	2.52	1.1
#39	IgG	1	4.54	1.22	3.72	2.22
#40	IgA	1	4.54	2	2.27	1.1
#41	IgG	4	4.54	1.32	3.44	0.63
#42	IgG	3	4.52	1.84	2.46	1.14
#43	IgA	3	4.45	1.6	2.78	1.31
#44	IgG	3	4.43	2.28	1.94	1.26
#45	IgG	1	4.42	1.27	3.48	0.71
#46	light chain	2	4.38	2.12	2.07	1.27
#47	IgA	10	4.27	1.75	2.44	1.37
#48	IgG	2	4.21	1.29	3.26	1.11
#49	IgA	2	4.18	1.53	2.73	1.01

#50	light chain	4	4.16	1.43	2.91	0.92
#51	IgG	6	4.12	1.7	2.42	0.95
#52	IgG	5	4.05	1.48	2.74	0.92
#53	IgA	2	4.04	1.96	2.06	1.41
#54	IgA	4	3.92	1.12	3.50	0.96
#55	IgG	1	3.91	1.29	3.03	1.82
#56	light chain	4	3.88	1.55	2.50	0.69
#57	light chain	3	3.84	2.06	1.86	1.27
#58	IgG	3	3.84	2.41	1.59	1.36
#59	IgA	2	3.81	1.11	3.43	0.64
#60	IgG	1	3.81	2.46	1.55	1.39
#61	IgG	4	3.66	1.67	2.19	1.22
#62	IgG	3	3.58	1.65	2.17	1.09
#63	IgG	1	3.53	1.64	2.15	1.48
#64	IgA	4	3.41	1.74	1.96	1.15
#65	IgA	7	3.38	2.29	1.48	1.48
#66	IgG	8	3.32	1.67	1.99	0.92
#67	light chain	3	3.19	1.12	2.85	1.11
#68	light chain	3	3.17	2.01	1.58	1.17
#69	light chain	8	3.13	0.87	3.60	0.8
#70	light chain	3	3.12	2.06	1.51	1.41
#71	IgA	2	3.07	0.95	3.23	0.84
#72	IgG	4	3.02	1.69	1.79	1.57
#73	IgA	5	2.96	1.68	1.76	1.37
#74	IgG	5	2.94	1.24	2.37	0.91
#75	IgG	1	2.83	1.03	2.75	0.73
#76	IgG	4	2.82	1.89	1.49	1.32
#77	IgA	3	2.77	1.23	2.25	0.62
#78	IgG	3	2.7	2.04	1.32	1.03
#79	IgG	5	2.38	1.25	1.90	1.27
#80	n/a	9	2.34	0.54	4.33	0.53
#81	light chain	n/a	2.25	1.48	1.52	0.9
#82	light chain	5	2.18	1.32	1.65	0.8
#83	IgA	6	2.06	1.3	1.58	1.56
#84	IgA	2	1.95	0.84	2.32	0.52
#85	light chain	7	1.94	1.32	1.47	0.94
#86	IgA	2	1.61	1.05	1.53	0.65
#87	light chain	6	1.43	0.8	1.79	0.6

SUV: Standardized uptake value; MM: multiple myeloma; TBR: spleen-to-bloodpool ratio

SUPPLEMENTARY TABLE S2: Summary of the treatment history of each patient

Patient No. #	MM Type	Prior therapy lines	Prior ASCT	Details of chemotherapeutic regimens
#1	light chain	2	yes	PAD-Rev, HDM/ASCT; Porm-PAD-Dara
#2	light chain	2	no	VCD; Pom-PAD-Dara
#3	IgG	1	yes	VCD, HDM/ASCT
#4	IgG	4	yes	PAD, HDM/ASCT; KRd; phase I study drug; PomPd
#5	light chain	1	yes	VCD, HDM/ASCT
#6	light chain	1	yes	PAD, HDM/ASCT
#7	IgA	2	yes	PAD-Rev, HDM/ASCT; Pom-PAD
#8	light chain	2	yes	PAD, HDM/ASCT; PAD-Rev
#9	light chain	3	yes	RAD, HDM/ASCT; VCD; BVD
#10	IgG	6	yes	RAD, HDM/ASCT; Vd; ERd; Pom-AD; Da-mono; Pom-AD-Dara
#11	IgG	4	yes	VRD-PACE, HDM/ASCT; PAT-SM6; PAD-Rev; Pom-PAD
#12	light chain	1	yes	PAD, HDM/ASCT
#13	IgG	2	yes	VRD-PACE, HDM/ASCT; PAT-SM6
#14	IgG	1	yes	PAD, HDM/ASCT
#15	IgG	2	yes	PAD, HDM/ASCT; KRd
#16	light chain	4	no	Vd; Rd; Pom-PAD; Pom-VCD
#17	IgG	1	yes	VCD, HDM/ASCT
#18	IgG	2	yes	Rd, HDM/ASCT; *RAD*
#19	IgG	1	yes	PAD, HDM/ASCT
#20	IgG	2	yes	VRD, HDM/ASCT; DaraRd
#21	light chain	2	yes	PAD, HDM/ASCT; VCD
#22	IgG	1	yes	VCD, HDM/ASCT
#23	IgA	4	yes	RAD, HDM/ASCT; VTD-PACE; KRd; EPd
#24	IgA	3	yes	PAD-Rev, HDM/ASCT; Pom-PAD; Pom-PAD-Dara
#25	IgG	3	yes	RAD, HDM/ASCT; DaraVd; Dexa-BEAM
#26	IgG	4	yes	VCD, HDM/ASCT; Vd; RAD; Pom-AD
#27	light chain	1	yes	VCD, HDM/ASCT
#28	IgG	3	yes	VCD, HDM/ASCT; KCd; KRd
#29	light chain	5	yes	VCD, HDM/ASCT; Rd; VCD; KRd; Da-mono
#30	IgG	4	yes	VRD, HDM/ASCT; PomCyD; KRd; DaraRd
#31	IgA	2	yes	VCD, HDM/ASCT; KRd
#32	IgG	2	yes	PAD, HDM/ASCT; VCD
#33	IgG	1	yes	PAD-Rev, HDM/ASCT
#34	IgG	3	yes	VCD, HDM/ASCT; Pom-PAD; Pom-PAD-Dara
#35	IgG	4	yes	RAD, HDM/ASCT; Vd, PAN-Vd; VRCD
#36	IgA	2	yes	VCD, HDM/ASCT; VRD

#37	light chain	2	yes	PAD, HDM/ASCT; Rd
#38	light chain	5	yes	PAD, HDM/ASCT; Rd; PAN-Vd; Da-mono; DaraPd
#39	IgG	1	yes	PAD-Rev, HDM/ASCT
#40	IgA	1	yes	VCD, HDM/ASCT
#41	IgG	4	yes	RAD, HDM/ASCT; Pom-PAD; KCd; KRd
#42	IgG	3	yes	VCD, HDM/ASCT; KRd; Pom-PAD
#43	IgA	3	yes	VCD, HDM/ASCT; KRd; Pom-PAD
#44	IgG	3	yes	RAD, HDM/ASCT; VRCD; BVD
#45	IgG	1	yes	VCD, HDM/ASCT
#46	light chain	2	no	VCD; RAD
#47	IgA	10	yes	PAD, HDM/ASCT; Rd; BRD; VD-PACE; Vd, Pd; RCD; KRd; PAD; DCEP
#48	IgG	2	yes	VCD, HDM/ASCT; PAD-Rev
#49	IgA	4	yes	VCD, HDM/ASCT; Rd; Pom-PAD; Dara-VCD
#50	light chain	6	yes	PAD, HDM/ASCT; Rd; BVD; Da-mono; DaraVd; Pom-PAD
#51	IgG	5	yes	VRD-PACE, HDM/ASCT; Pom-PAD; VRCD; PomVd; Dara-KPd
#52	IgG	2	yes	VCD, HDM/ASCT; RAD
#53	IgA	4	yes	VCD, HDM/ASCT; PVd; Pom-PAD-Dara, Dara-Kd
#54	IgA	1	yes	VCD, HDM/ASCT
#55	IgG	4	yes	VCD, HDM/ASCT; RAD; VRD-PACE; Dexa-BEAM
#56	light chain	3	no	VCD; VTD-PACE; VRD
#57	light chain	3	yes	VRD, HDM/ASCT; KRd
#58	IgG	2	yes	VCD, HDM/ASCT; RAD
#59	IgA	1	yes	VCD, HDM/ASCT
#60	IgG	4	yes	VRD, HDM/ASCT; Dexa-BEAM; PAD; Pom-PAD
#61	IgG	3	yes	Vd, HDM/ASCT; Rd; PAD-Rev
#62	IgG	1	yes	RAD, HDM/ASCT
#63	IgG	4	yes	VCD, HDM/ASCT; RAD; KRd; DaraVd
#64	IgA	7	yes	VCD, HDM/ASCT; PAN-Vd; VRD; Pd; KCd; PAD; Da-
#65	IgA	8	yes	PAD, HDM/ASCT; VCD; PAD; Rd; PAN-*Vd*; VDT-PACE; DaraPd; Dara-KPd
#66	IgG	3	yes	PAD, HDM/ASCT; VCD; PAD
#67	light chain	3	yes	PAD, HDM/ASCT; PAD-Thal; VRD
#68	light chain	8	yes	PAD, HDM/ASCT; Vd; Rd; BVD; PAD; Dexa-BEAM; Pom-PAD; KCd
#69	light chain	3	yes	PAD, HDM/ASCT; VCD; Pom-PAD
#70	light chain	2	yes	PAD, HDM/ASCT; VCD
#71	IgA	4	yes	VCD, HDM/ASCT; Rd; VTD-PACE; VRD-PACE
#72	IgG	5	yes	VMP, HDM/ASCT; VRD-PACE; Dexa-BEAM; PAT-SM6; KRd
#73	IgA	5	yes	PAD, HDM/ASCT; VCD; Rd; Pd; Rd

#74	IgG	1	yes	VCD, HDM/ASCT
#75	IgG	4	yes	VCD, HDM/ASCT; KRd; Kd; DaraRd
#76	IgG	3	yes	RAD, HDM/ASCT; Vd; Pom-PAD
#77	IgA	3	n/a	PAD-Rev; Da-mono; KRd
#78	IgG	5	yes	VMP; HDM/ASCT; VCD; Rd; VCD; PAD-Rev
#79	IgG	9	yes	RAD, HDM/ASCT; VCD, BVD; VRCD; Rd; RAD; VRCD; KCd; Dexa-BEAM
#80	n/a	n/a	yes	n/a
#81	light chain	5	no	PAD, Rd; PAD; RCD; Pom-PAD
#82	light chain	6	yes	VCD, HDM/ASCT; Rd; Vd; Pd; KCd; Da-mono
#83	IgA	2	yes	VRD, HDM/ASCT; DaraKd
#84	IgA	4	no	Vd; VMP; TD; Rd
#85	light chain	7	yes	Vd, HDM/ASCT; Rd; Pd; VTD; VTD-PACE; VRD; DCEP
#86	IgA	2	yes	VRD, HDM/ASCT; VTD-PACE
#87	light chain	6	yes	VCD, HDM/ASCT; VTD-PACE; Pd, BRD; Vd

PAD-Rev: bortezomib, adriamycin, dexamethasone, lenalidomide; HDM/ASCT: high-dose melphalan, autologous stem cell transplantation; Pom-PAD-Dara: pomalidomide, bortezomib, doxorubicin, dexamethasone, daratumumab; VCD: velcade, cyclophasphamide, dexamethasone; PAD: bortezomib, doxorubicin, dexamethasone; KRd: carfilzomib, lenalidomide, dexamethasone; RAD: lenalidomide, doxorubicin, dexamethasone; VRD-PACE: bortezomib, lenalidomide, dexamethasone, cisplatin, adriamycin, cyclophosphamide, etoposide; VD-PACE: bortezomib, dexamethasone, cisplatin, adriamycin, cyclophosphamide, etoposide; VTD-PACE: bortezomib, thalidomide, dexamethasone, cisplatin, adriamycin, cyclophosphamide, etoposide; Vd: bortezomib, dexamethasone; Rd: lenalidomide, dexamethasone; Pd: pomalidomide, dexamethasone; VRCD: bortezomib, lenalidomide, cyclophosphamide, dexamethasone; BVD: bendamustine, bortezomib, dexamethasone; BRD: bendamustine, lenalidomide, dexamethasone; VMP: bortezomib, melphalan, prednisolone; TD: thalidomide, dexamethasone; VRD: bortezomib, lenalidomide, dexamethasone; Kd: carfilzomib, dexamethasone; CD: cyclophosphamide, dexamethasone; RCD: lenalidomide, cyclophosphamide, dexamethasone; Da-*mono: daratumomab monotherapy;* DaraRd: daratumomab, lenalidomide, dexamethasone; Dexa-BEAM: dexamethasone, carmustine, cytarabine, etoposide, melphalan; KCd: Carfilzomib, cyclophosphamide, and dexamethasone; PAD-Thal: bortezomib, adriamycin, dexamethasone, thalidomide; DaraPd: daratumumab, pomalidomide, dexamethasone; Dara-KPd: daratumumab, carfilzomib, pomalidomide, dexamethasone; PAN-Vd: panobinostat, bortezomib, dexamethasone; DaraVd: daratumumab, bortezomib, dexamethasone; Dara-VCD: daratumumab, velcade, cyclophasphamide, dexamethasone; DCEP: dexamethasone, cyclophosphamide, etoposide, cisplatin; EPd: elotuzumab, pomalidomide, dexamethasone; VTD: bortezomib, thalidomide, dexamethasone; PomCyD: pomalidomide, cyclophosphamide, dexamethasone; KRd: carfilzomib, lenalidomide, dexamethasone; DaraVd: daratumumab, bortezomib, dexamethasone; DaraRd: Daratumumab, revlimid, dexamethasone; ERd: elotuzumab, lenalidomide, dexamethasone; Pom-VCD: pomalidomide, bortezomib, cyclophosphamide; dexamethasone; PomVD: pomalidomide, bortezomib, dexamethasone; DaraKd: daratumumab, carfolzomib, dexamethasone; n/a not available

SUPPLEMENTARY FIGURE S1:

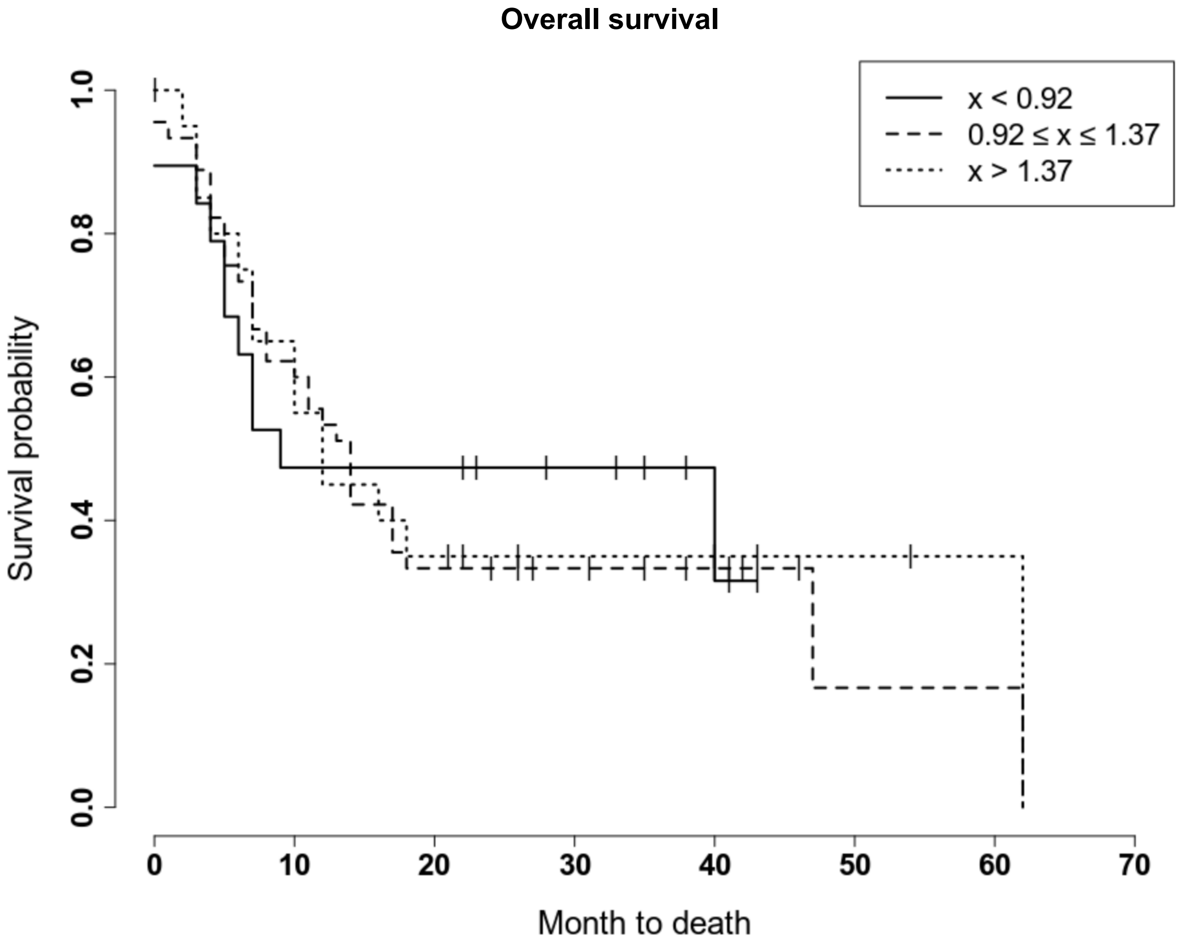

Supplementary Figure S1: Prognostic value of liver ^{68}Ga-Pentixafor uptake. ^{68}Ga-Pentixafor uptake of the liver has no predictive value and is not related to the number of prior treatment lines. Given are the cumulated survival (y-axis) and the overall survival (in months; x-axis).
